# Consumption of sweet foods and mammographic breast density: a cross-sectional study

**DOI:** 10.1186/1471-2458-14-554

**Published:** 2014-06-26

**Authors:** Caroline S Duchaine, Isabelle Dumas, Caroline Diorio

**Affiliations:** 1 Département de médecine sociale et préventive, Centre de recherche sur le cancer, Université Laval, 2325, rue de l’Université, G1V 0A6 Quebec City, QC, Canada; 2 Axe Oncologie, Centre de recherche du CHU de Québec, Hôpital du Saint-Sacrement, 1050, chemin Ste-Foy, G1S 4L8 Quebec City, QC, Canada; 3 Centre des maladies du sein Deschênes-Fabia, Hôpital St-Sacrement du CHU de Québec, 1050, chemin Ste-Foy, G1S 4L8 Quebec, QC, Canada

**Keywords:** Epidemiological, Breast density, Sugar intake, Breast cancer, Diet

## Abstract

**Background:**

The increasing consumption of sugar worldwide seems to lead to several health problems, including some types of cancer. While some studies reported a positive association between sweet foods intake and breast cancer risk, little is known about their relation to mammographic density (MD), a strong breast cancer risk factor. This study examined the association of sweet foods and drinks intake with MD among 776 premenopausal and 779 postmenopausal women recruited at mammography.

**Methods:**

A food-frequency questionnaire was used to assess intake of sweet foods, sugar-sweetened beverages and spoonsful of sugar added. Percent and absolute breast density were estimated using a computer-assisted method. Multivariate generalized linear models were used to evaluate associations. All models were adjusted for potential confounders, including age and body mass index.

**Results:**

For increasing quartiles of sugar-sweetened beverages intake, adjusted-mean absolute density was respectively 32, 34, 32 and 36 cm^2^ among all women (*P*_trend_ = 0.040) and 43, 46, 44 and 51 cm^2^ among premenopausal women (*P*_trend_ = 0.007). For increasing quartiles of sweet foods intake, adjusted-mean percent density was respectively 16, 16, 17 and 19% among postmenopausal women (*P*_trend_ = 0.036). No association was shown between intake of spoonsful of sugar added and MD.

**Conclusion:**

Our results suggest that an increase in sweet foods or sugar-sweetened beverage intake is associated with higher MD.

## Background

It has been recently suggested that consumption of foods high in added sugar is the source of many of the greatest health problems in the world today [1]. Sugar consumption has tripled over the past 50 years and the World Health Organization (WHO), in collaboration with the FAO (Food and Agriculture Organization of the United Nations), has published some recommendations to reduce this consumption worldwide [2]. In fact, excessive sugar intake seems to lead to human health problems like the ones associated with metabolic syndrome [2,3] and several types of cancer, including breast cancer [4-6]. At a cellular level, it has been shown that sugar would enhance cellular proliferation and migration, induce DNA damage and increase inflammation [7,8]. All of these adverse effects are related to cancer pathogenesis.

Several studies [4,9-20], although not all [21-29], have found a positive association between sweet foods/drinks and breast cancer risk among premenopausal women [14,16,20], postmenopausal women [16,20] or both combined [4,9-13,15,17-20]. In all of the studies that found a positive association, the frequencies of consumption of several sweet foods or drinks were summed into one [9,11,12,14-19] or two [4,10,13,20] groups. In contrast, among the studies that showed no association, most of them have examined some sweet foods and drinks separately [21,23-25,27-29]. These results may suggest that the sum of sweet items consumed would be associated with breast cancer risk rather than a specific sweet food item intake, therefore it would be the amount of sweet items intake that has an effect, and not a specific food.

Mammographic density (MD) is a well-established breast cancer risk factor and it appears to act as an intermediate marker [30-32]. Considering that elevated MD may reflect an increase in the proliferation of fibroglandular breast cells [33], it is likely that a high intake of sweet foods and sugar-sweetened beverages may lead to an increase in MD. Up to now, two studies conducted among pre- and postmenopausal women have evaluated the association between consumption of sweet items and MD [34,35]. Voon *et al.* observed a positive association between intake of sweet foods and MD [34], while Masala *et al.* found a non-significant positive association between intake of spoonsful of sugar added and MD [35].

The present study aimed at clarifying the relation of intake of sweet foods, sugar-sweetened beverages and spoonsful of sugar added to beverages and foods with MD among premenopausal and postmenopausal women.

## Methods

### Study population and recruitment procedures

While study population and recruitment procedures have been described elsewhere [36,37], here is a summary. Women of the study population have been recruited among those who received a screening mammogram between February 2001 and March 2002 in two private radiology clinics in Quebec City. Eligible women had to be classified as premenopausal or postmenopausal according to the Nurses’ Health Study criteria [38], have no personal history of cancer or breast surgery, have no endocrine diseases, have never taken selective estrogen-receptor modulators, not be pregnant, and have not used exogenous hormones in the three months preceding the mammography. Of the 1574 women who have participated in the original study, we excluded 11 women who did not completed the food frequency questionnaire (FFQ), five women who followed a special diabetic diet and three women who reported extremely high (>5000 kcal/day) or extremely low (<600 kcal/day) caloric intake for the purpose of the present study. Thus the present analysis includes 1555 women (776 premenopausal and 779 postmenopausal). The study was approved by the Research Ethics Review Board-Hôpital Saint-Sacrement at the CHU de Québec. All study participants provided written informed consent.

### Data collection

Height, weight, waist and hip circumferences were measured by a trained nurse at recruitment. Information on breast cancer risk factors and physical activity were collected during a phone interview performed by trained interviewers. Physical activity was assessed using the Nurses’ Health Study II Activity and Inactivity Questionnaire [39] and then expressed as metabolic equivalent (MET) hours per week.

### Diet assessment

Data on diet were collected with a 161-item, validated self-administered semi-quantitative FFQ (97GP copyrighted at Harvard University, Boston, MA) [40,41]. A standard serving size was assigned for each item and its frequency of consumption over the previous year was selected between never, less than one serving per month, 1–3 servings per month, one serving per week, 2–4 servings per week, 5–6 servings per week, one serving per day, 2–3 servings per day or more than three servings per day. From this FFQ, 14 items were classified as sweet foods (ice cream, chocolate, candy with chocolate, candy, homemade cookie, commercial cookie, brownie, donut, homemade cake, commercial cake, homemade pie, commercial pie, other homemade pastries and other commercial pastries), four items as sugar-sweetened beverages (cola with sugar, cola with sugar but caffeine free, other carbonated beverage with sugar and sweet fruit juice) and two items as spoonsful of sugar added to beverages and foods (tablespoon of jam, syrup or honey and teaspoon of sugar used).

### Digitization of mammograms and assessment of mammographic density

Mammograms were digitized using a Kodak LS-85 laser film scanner. All mammograms were blindly evaluated by a trained reader within one month of each other. MD was assessed using interactive threshold Cumulus software, a computer assisted method [30]. A craniocaudal view of the right or left breast was randomly selected and MD was measured for each participant in batches of 100 images. Two measurements of MD were obtained: the proportion of the breast showing tissue density (percent density in %) and the absolute amount of dense tissue (absolute density in cm^2^). Duplicates of images were introduced in each batch in order to evaluate reproducibility of measurement. The intra-class correlation coefficients were 0.98 and 0.98 and the inter-group coefficients of variation were 4% and 5% for percent and absolute density, respectively.

### Statistical analysis

The frequency of consumption of each sweet item was transformed in number of servings per week, using the middle of each category when necessary. We created four groups of foods by adding the number of servings per week of 1- all 14 sweet foods, 2- all four sugar-sweetened beverages, 3- the two items related to spoonsful of sugar added and 4- all items aforementioned. Partial Spearman correlations between the number of servings per week and MD as continuous variables were estimated. As we found no specific categorization in the literature and that most of studies on sweet foods and breast cancer risk have used percentile categorization, the number of servings per week of each group was categorized in quartiles by using all women. Percent and absolute density were square root-transformed except for percent density among premenopausal women, in order to obtain a normal distribution of these variables. Then, multivariate-adjusted means of MD by quartile were estimated using generalized linear models, and the linear trend across quartiles was tested by using linear contrast with the median of servings per week within each of the quartiles as the value. Means of MD are presented as back-transformed values. For statistically significant results, additional multivariate-adjusted models were performed using categorizations of servings per week that are more useful for public health purposes. Potential confounders identified *a priori* and included in the adjusted models and correlations were age at mammography (year), body mass index (BMI, kg/m^2^), age at menarche (years), age at first full-term pregnancy (years), number of full-term pregnancies, duration of breastfeeding (months), duration of use of oral contraceptive (years), duration of use of hormonal therapy (years), alcohol intake (servings/week), family history of breast cancer (yes/no), number of biopsies, smoking status (nonsmoker, ex-smoker, current smoker), education (highest degree completed: primary or less, secondary, college, university), waist-to-hip ratio, physical activity (MET-h/week), calorie intake (kcal/day) and menopausal status (premenopausal, postmenopausal) when applicable. Further adjustment for intake of calcium (mg/day), intake of vitamin D (IU/day), season at time of mammography (winter, spring, summer, fall) and use of multivitamin-multimineral supplements (never, past, current user) had little or no influence on the estimates. Therefore, they were not added in the models.

Because the possible modifying effect of BMI and physical activity was reported in the literature, we also conducted exploratory analyses evaluating partial Spearman correlations of intake of sugar-sweetened beverages and sweet foods (the two groups that presented a statistically significant correlation with MD) with MD stratified by BMI (≤25 and >25 kg/m^2^) and by physical activity (≤median (21 MET-h/wk) and >median). All tests were 2-sided and a p-value < 0.05 was considered statistically significant. All statistical analyses were carried out using the SAS (SAS Institute, Inc., Cary, NC) software system.

## Results

### Study population

Characteristics of the 1555 women included in the analyses are described in Table 1. In summary, the mean age of the women was 54.1 years, with a mean of 46.7 years for premenopausal women and 61.4 years for postmenopausal women. As expected, postmenopausal women had lower percent and absolute densities compared with premenopausal women (18.5 versus 42.0% and 23.3 versus 46.5cm^2^ respectively). On average, postmenopausal women consumed more sweet foods (11.8 versus 7.7 servings per week), spoonsful of sugar added (8.2 versus 6.9 servings per week) and all sweet items grouped (21.9 versus 16.8 servings per week) than premenopausal women, with a higher total caloric intake per day. The frequency of sugar-sweetened beverages intake was similar in both groups (1.9 versus 2.1 servings per week for postmenopausal and premenopausal women respectively).

**Table 1:** Characteristics of the study population

	**Total n = 1555**	Premenopausal n = 776*	Postmenopausal n = 779**
Age (years), mean (SD)	54.1 (9.4)	46.7 (4.6)	61.4 (6.8)
Age at 1^st^ full-term pregnancy (years), mean (SD)^a^	25.7 (4.2)	26.3 (4.2)	25.2 (4.1)
Body mass index (kg/m^2^), mean (SD)	26.1 (4.7)	25.2 (4.5)	27.1 (4.7)
Waist-to-hip ratio, mean (SD)	0.8 (0.1)	0.8 (0.1)	0.8 (0.1)
Age at menarche (years), mean (SD)	12.7 (1.6)	12.8 (1.6)	12.7 (1.6)
Alcohol intake (drinks/week), mean (SD)	3.0 (4.1)	3.4 (3.8)	2.5 (4.4)
Physical activity (MET-h/week), mean (SD)	26.3 (22.8)	27.0 (22.3)	25.7 (23.4)
Caloric intake (kcal/day), mean (SD)	1941.1 (585.8)	1911.5 (521.4)	1970.6 (642.5)
Percent density (%), mean (SD)	30.2 (24.0)	42.0 (24.4)	18.5 (16.8)
Absolute density (cm^2^), mean (SD)	34.9 (27.7)	46.5 (28.7)	23.3 (21.0)
Sugar-sweetened beverages^b^ (servings/week), mean (SD)	2.0 (4.0)	2.1 (4.4)	1.9 (3.5)
Sweet foods^c^ (servings/week), mean (SD)	9.8 (9.7)	7.7 (7.6)	11.8 (11.0)
Spoonsful of sugar added^d^ (servings/week), mean (SD)	7.6 (11.0)	6.9 (11.0)	8.2 (10.9)
Total of all sweet items (servings/week), mean (SD)	19.3 (17.4)	16.8 (15.3)	21.9 (18.8)
Parity (yes), n (%)	1167 (75.1)	584 (75.3)	583 (74.8)
Breastfeeding (yes), n (%)^a^	532 (45.6)	363 (62.2)	169 (29.0)
Use of hormonal therapy (yes), n (%)	347 (22.4)	45 (5.8)	302 (39.1)
Use of oral contraceptive (yes), n (%)	1128 (72.7)	712 (91.9)	416 (53.5)
Family history of breast cancer (yes), n (%)	521 (33.5)	284 (36.6)	237 (30.4)
Breast biopsy (yes), n (%)	238 (15.3)	112 (14.4)	126 (16.2)
Education (highest degree completed), n (%)			
Primary or less	255 (16.4)	51 (6.6)	204 (26.2)
Secondary	510 (32.8)	242 (31.2)	268 (34.4)
College	304 (19.6)	206 (26.6)	98 (12.6)
University	486 (31.3)	277 (35.7)	209 (26.8)
Smoking status, n (%)			
Nonsmoker	811 (52.2)	354 (45.6)	457 (58.7)
Ex-smoker	524 (33.7)	306 (39.4)	218 (28.0)
Current smoker	220 (14.1)	116 (15.0)	104 (13.4)

*Missing values for sweet foods (n = 2), sugar-sweetened beverages (n = 5) and total of all sweets (n = 7).

**Missing values for sweet foods (n = 11), sugar-sweetened beverages (n = 2), spoonsful of sugar added (n = 1) and total of all sweets (n = 14).

^a^Among parous women.

^b^Including cola with sugar, cola with sugar but caffeine free, other carbonated beverage with sugar and sweet fruits juice.

^c^Including ice cream, chocolate, candy with chocolate, candy, homemade cookie, commercial cookie, brownie, donut, homemade cake, commercial cake, homemade pie, commercial pie, other homemade pastries and other commercial pastries.

^d^Including teaspoons of sugar added to beverages and foods and tablespoons of jam, honey and syrup.

*Abbreviations*: *SD* standard deviation, *MET-h/wk* Metabolic equivalent hours per week.

### Consumption of sugar-sweetened beverages

**Figure 1:**
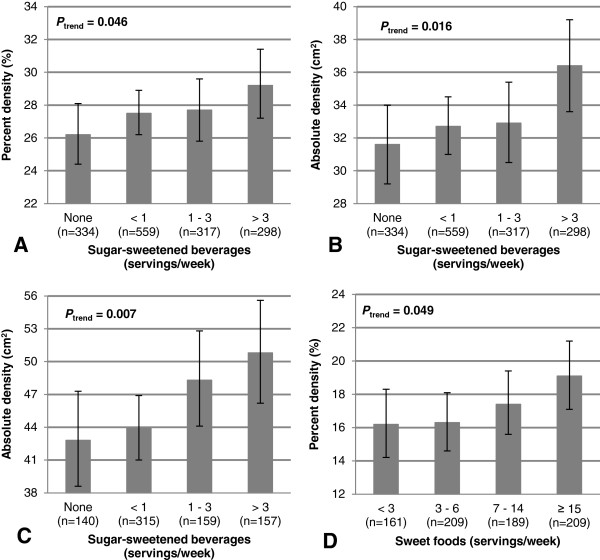
**Mammographic density according to number of servings per week of beverages or sweet foods.** Percent **(A)** and absolute **(B)** density according to weekly servings of sugar-sweetened beverages among all women. Absolute density according to weekly servings of sugar-sweetened beverages among premenopausal women **(C)**. Percent density according to weekly servings of sweet foods among postmenopausal women **(D)**. Percent and absolute density were square root-transformed for all analyses, except for percent density among premenopausal, to obtain an approximate normal distribution. Means are presented as back-transformed values for these analyses. Error lines represent the 95% confidence intervals. All analyses were adjusted for total caloric intake, age at mammography, BMI, waist-to-hip ratio, age at menarche, number of full-term pregnancies, age at first full-term pregnancy, duration of use of hormonal contraceptive, duration of use of hormonal therapy, alcohol intake, physical activity, family history of breast cancer, number of biopsies, smoking status, education, breastfeeding and menopausal status when applicable. *P*_trend_ = p-value for linear contrast test.

The associations between consumption of sugar-sweetened beverages and MD are shown in Table 2. Among all women, ascending quartiles of sugar-sweetened beverages intake was positively associated with percent density (*P*_*trend*_ = 0.17, *r* = 0.054 (*P* = 0.038)) and absolute density (*P*_*trend*_ = 0.040, *r* = 0.051 (*P* = 0.049)). For increasing weekly servings of sugar-sweetened beverages (none, <1, 1–3 and >3), MD was respectively 26.2, 27.5, 27.7, 29.2% (*P*_*trend*_ = 0.046, Figure 1A) and 31.6, 32.7, 32.9, 36.4 cm^2^ (*P*_*trend*_ = 0.016, Figure 1B). When stratified by menopausal status, the positive association between sugar-sweetened beverages intake and absolute density remained significant among premenopausal (*P*_*trend*_ = 0.007, *r* = 0.088 (*P* = 0.016)) but not among postmenopausal women (*P*_*trend*_ = 0.96, *r* = 0.013 (*P* = 0.74)). For increasing weekly servings of sugar-sweetened beverages (none, <1, 1-3 and >3), absolute density was respectively 42.8, 43.9, 48.3 and 50.8 cm^2^ among premenopausal women (*P*_*trend*_ = 0.007, Figure 1C).

**Table 2:** **Associations**^**a **^**of consumption of sweets with mammographic density among all women and by menopausal status**

Quartiles* of servings per week	**All women**			**Premenopausal women**			**Postmenopausal women**		
		**Percent density**	**Absolute density**		**Percent density**	**Absolute density**		**Percent density**	**Absolute density**
	n**	**% [95% CI]**^**a**^	**cm**^**2 **^**[95% CI]**^**a**^	n**	**% [95% CI]**^**a**^	**cm**^**2 **^**[95% CI]**^**a**^	n**	**% [95% CI]**^**a**^	**cm**^**2 **^**[95% CI]**^**a**^
**Sugar-sweetened beverages**^**b**^									
0	334	26.3 [24.4-28.1]	31.6 [29.3-34.0]	140	41.8 [38.4-45.1]	42.8 [38.6-47.3]	194	16.6 [14.8-18.5]	22.7 [20.2-25.4]
0.01-0.43	391	28.5 [26.8-30.2]	34.1 [31.9-36.4]	210	42.5 [39.7-45.2]	45.5 [41.9-49.4]	181	18.8 [16.9-20.9]	24.2 [21.6-27.0]
0.44-1.69	433	26.8 [25.2-28.4]	31.5 [29.5-33.5]	220	40.4 [37.8-43.1]	43.9 [40.4-47.4]	213	17.0 [15.3-18.8]	21.1 [18.9-23.4]
>1.69	390	28.8 [27.0-30.7]	35.6 [33.3-38.1]	201	44.0 [41.2-47.0]	51.1 [47.0-55.4]	189	17.1 [15.2-19.1]	22.9 [20.3-25.6]
*P*_trend_		0.172	**0.040**		0.240	**0.007**		0.731	0.958
Spearman *r* (*P*)		**0.054 (0.038)**	**0.051 (0.049)**		0.049 (0.185)	**0.088 (0.016)**		0.026 (0.492)	0.013 (0.735)
**Sweet foods**^**c**^									
0-2.95	380	27.9 [26.1-29.8]	33.9 [31.6-36.4]	219	43.5 [40.7-46.4]	47.4 [43.6-51.4]	161	16.2 [14.2-18.3]	22.2 [19.5-25.1]
2.96-6.79	390	26.7 [25.1-28.5]	32.1 [29.9-34.3]	231	42.2 [39.6-44.7]	45.2 [41.7-48.7]	159	15.9 [14.0-17.9]	21.0 [18.4-23.8]
6.80-11.90	383	28.1 [26.4-29.9]	34.4 [32.2-36.7]	195	43.0 [40.2-45.9]	48.9 [44.9-53.0]	188	17.4 [15.6-19.3]	22.7 [20.3-25.3]
>11.90	389	27.9 [26.1-29.9]	32.7 [30.3-35.2]	129	38.8 [35.1-42.5]	41.2 [36.6-46.1]	260	18.8 [17.0-20.7]	23.7 [21.3-26.1]
*P*_trend_		0.717	0.748		0.065	0.077		**0.036**	0.266
Spearman *r* (*P*)		0.008 (0.772)	-0.006 (0.829)		-0.052 (0.161)	-0.044 (0.233)		**0.073 (0.048)**	0.043 (0.242)
**Spoonsful of sugar added**^**d**^									
0-0.56	428	27.0 [25.4-28.7]	32.5 [30.4-34.6]	234	42.1 [39.4-44.7]	44.2 [40.7-47.8]	194	17.2 [15.3-19.1]	23.0 [20.5-25.7]
0.57-3.01	392	26.7 [25.0-28.4]	32.3 [30.2-34.6]	211	41.5 [38.8-44.2]	46.2 [42.6-50.0]	181	16.4 [14.6-18.4]	21.3 [18.9-23.9]
3.02-9.31	327	28.6 [26.7-30.5]	34.2 [31.8-36.7]	148	41.8 [38.6-45.0]	46.3 [41.9-50.8]	179	18.2 [16.2-20.2]	23.3 [20.8-26.1]
>9.31	407	28.4 [26.7-30.2]	34.2 [32.0-36.5]	183	43.4 [40.4-46.3]	48.1 [44.1-52.3]	224	17.5 [15.8-19.4]	22.8 [20.5-25.3]
*P*_trend_		0.164	0.211		0.415	0.208		0.594	0.807
Spearman *r* (*P*)		0.030 (0.241)	0.026 (0.311)		0.034 (0.362)	0.055 (0.138)		0.016 (0.661)	0.003 (0.929)
**Total of all sweet items**									
0-7.14	380	26.2 [24.5-28.1]	31.7 [29.4-34.1]	219	41.3 [38.5-44.0]	43.5 [39.9-47.3]	161	16.0 [14.0-18.2]	21.8 [19.0-24.8]
7.15-14.0	383	29.1 [27.4-31.0]	34.9 [32.7-37.3]	213	45.5 [42.8-48.2]	49.3 [45.6-53.2]	170	17.9 [15.9-20.0]	23.1 [20.5-25.9]
14.01-26.25	384	27.7 [25.9-29.4]	33.4 [31.2-35.7]	188	40.2 [37.3-43.1]	46.1 [42.2-50.2]	196	17.8 [16.0-19.8]	22.8 [20.4-25.4]
>26.25	387	27.6 [25.8-29.5]	32.9 [30.6-35.4]	149	41.5 [38.0-45.0]	44.7 [40.2-49.6]	238	17.4 [15.6-19.3]	22.7 [20.3-25.2]
*P*_trend_		0.740	0.909		0.503	0.830		0.536	0.794
Spearman *r* (*P*)		0.026 (0.311)	0.017 (0.513)		0.001 (0.984)	0.019 (0.609)		0.044 (0.238)	0.019 (0.604)

^a^All analyses (linear models and Spearman correlations) were adjusted for: Total caloric intake per day, age at mammography, body mass index, waist-to-hip ratio, menopausal status (for total women only), age at menarche, parity, number of full-term pregnancies, age at first full-term pregnancy, duration of use of hormonal contraceptive, duration of use of hormonal therapy, alcohol intake, physical activity, family history of breast cancer, number of breast biopsies, smoking status, education and breastfeeding. Percent and absolute density were square root-transformed for all analyses, except for percent density among premenopausal, to obtain an approximate normal distribution. Means are presented as back-transformed values for these analyses.

^b^Including cola with sugar, cola with sugar but caffeine free, other carbonated beverage with sugar and sweet fruits juice.

^c^Including ice cream, chocolate, candy with chocolate, candy, homemade cookie, commercial cookie, brownie, donut, homemade cake, commercial cake, homemade pie, commercial pie, other homemade pastries and other commercial pastries.

^d^Including teaspoons of sugar added to beverages and foods and tablespoons of jam, honey and syrup.

*Quartiles are made using all women.

**Number do not add up because of missing data.

The statistically significant results are in bold.

*Abbreviations*: *CI* confidence interval, *r* correlation, *P*_*trend*_ p-value for linear contrast test.

### Consumption of sweet foods

The associations between consumption of sweet foods and MD are also shown in Table 2. Among postmenopausal women, ascending quartiles of sweet foods intake was positively associated with percent density (*P*_*trend*_ = 0.036, *r* = 0.073 (*P* = 0.048)). For increasing weekly servings of sweet foods (<3, 3–6, 7–14 and ≥15), percent density was respectively 16.2, 16.3, 17.4 and 19.1% (*P*_*trend*_ = 0.049, Figure 1D). However, we observed no association between sweet foods consumption and absolute density among postmenopausal women (*P*_*trend*_ = 0.27, *r* = 0.043 (*P* = 0.24)). Furthermore, the consumption of this group of foods was not associated with any measure of MD among all or premenopausal women.

### Consumption of spoonsful of sugar added and total of all sweet items

The consumption of spoonsful of sugar added and the total of all sweet items were not associated with MD among all women or within strata of menopausal status (Table 2).

### Stratified analysis by BMI and physical activity

Partial Spearman correlations of intake of sweet foods or sugar-sweetened beverages with percent or absolute density stratified by BMI or physical activity are detailed in Additional file 1. Regarding the positive correlation observed between the intake of sugar-sweetened beverages and MD among all women, this correlation remained significant only among the stratum of less active women for percent density (*r* = 0.080, *P* = 0.030) but not for absolute density (*r* = 0.060, *P* = 0.103). As to the correlation observed between sweet foods intake and percent density among postmenopausal women, this positive correlation remained significant only for those who are less active (*r* = 0.145, *P* = 0.005). No other significant correlation was observed within strata of BMI or physical activity.

## Discussion

In this study, we found that sugar-sweetened beverage intake was positively associated with MD among all women or limited to premenopausal women. We also found that the intake of sweet foods including mostly desserts was positively associated with MD in postmenopausal women. Among all women, those who had a sugar-sweetened beverages intake of more than three servings per week presented a 3% difference in percent density compared with those who did not drink this type of beverages (29.2% versus 26.2%). By comparison, it has been shown that healthy women at high risk of developing breast cancer who received tamoxifen for 54 months had a reduction of 6.4% in MD [42], and since tamoxifen can reduce the risk of breast cancer by 30-50% in high-risk women [43,44], an increase of about 3% in MD is not negligible in terms of breast cancer risk. In our population the consumption of sugar-sweetened beverages is not very high, but in the United States, a citizen consumes on average 12 servings of soda per week (1 serving = 1 bottle of 355 ml) of which 58% contain sugar [1].

To our knowledge, the association between consumption of sweet foods and MD has been assessed by one group other than ours [34]. In this cross-sectional study of 64 Malaysian women aged between 35 and 75 years, Voon and Chelliah analyzed the association between consumption of sweet foods summed in one group and MD divided in two groups (low: group I and II and high: group III and IV of the BI-RADS classification) and found a significant positive association [Odds ratio (OR) = 3.6, 95% CI (1.2-10.8)]. Unfortunately, there is no mention of which sweet foods were included in their group. Moreover, no stratified analysis by menopausal status was performed due to their small sample size. Several studies have examined the link between the consumption of desserts and breast cancer risk [4,9-13,19,20,22,25,26,45] and found a positive association [4,9-13,19,20]. Among them, only one had stratified their analyses by menopausal status [20] and one had stratified by age [4] which can serve as a proxy for menopausal status. Tavani *et al.*[4] found a positive association between dessert intake and breast cancer risk for all women and, when stratified by age, this association was significant only among women aged 60 years or more. In contrast, Bradshaw *et al.*[20] found a positive association between dessert intake and breast cancer risk among premenopausal and postmenopausal women with a slightly higher effect among premenopausal women. In our study, the positive association between sweet foods intake and MD is shown for postmenopausal only. Thus, our results suggested that a higher consumption of sweet foods may lead to higher MD and eventually higher breast cancer risk, at least for postmenopausal women. Since we observed no association between sweet foods intake and MD among premenopausal women, MD may not be the only pathway that could link sweet foods intake to breast cancer risk. The group of sweet foods is composed in majority of items that have a lot of sugar but also a lot of fat, and the consumption of fat has been positively associated with breast cancer risk and MD among postmenopausal in some studies [46-49]. However, the role of fat consumption in breast cancer risk or on MD is still controversial [49-52].

In a longitudinal study, Masala *et al.*[35] assessed the relation between consumption at baseline of a group of foods named sugar (that included jam, honey and sugar added to coffee, tea and yogurt) derived from a FFQ of 160 items and MD evaluated 5 years after enrollment according to Wolf’s classification (dichotomized in high (P2 + DY) and low (N1 + P1)). Their study population was composed of 1668 women aged 53 years on average and 62.4% were postmenopausal at baseline [35]. They found that women among the highest tertile of sugar consumption had higher MD, but the association was not statistically significant [OR = 1.22; 95% CI (0.92-1.61), *P* = 0.17]. In our population, we observed a similar tendency with the consumption of spoonsful of sugar added, which is comparable to their sugar group, which was not statistically significant, agreeing with their results. Unfortunately, among studies on breast cancer risk, none of them made an analysis with consumption of spoonsful of sugar added similar to ours. Nonetheless, a possible explanation for the lack of association between the consumption of spoonsful of sugar added and MD may be related to the composition of table sugar. Contrary to commercial desserts and sweet drinks which contain more grams of fructose than glucose, table sugar contains equal amounts of fructose and glucose [53]. Fructose and glucose are both simple sugars but they are absorbed and metabolized by different pathways in the organism and some authors have suggested that fructose could be more harmful than glucose to human health due to this difference [53,54]. It has been recently suggested that excessive consumption of fructose may increase breast cancer risk [7].

In the present study, we observed a positive association between sugar-sweetened beverages intake and MD among all women and particularly among premenopausal women. To our knowledge, this relationship was not assessed in another study. Nonetheless, our results are consistent with those from Witte and colleagues [14] who found a positive association between sweetened beverages intake and breast cancer risk among premenopausal women. Similarly, Potischman *et al.*[18] found a positive association with consumption of sweet items that included beverages, foods and spoonsful of sugar added among their study population composed of premenopausal women only. They evaluated the contribution of each component of that food group and found that the major contributor in percentage was regular cola soft drinks. However, the creation of a new group of full-sugar drinks (including all soft drinks caffeinated or not, and other fruit drinks with sugar) did not reveal any association. In contrast to the sweet foods group, the sugar-sweetened beverages group is composed of drinks mostly made of sugar (made of high-fructose corn syrup) and water suggesting that the positive association shown with the consumption of those drinks and MD or breast cancer risk among premenopausal women would be more related to the consumption of sugar, particularly fructose.

Stratified analyses by BMI and physical activity reveal that our association of sugar-sweetened beverages or sweet foods intake with MD is stronger among all less active women or among postmenopausal less active women respectively. Two breast cancer risk studies stratified their analyses by BMI and physical activity. One found that the association between intake of desserts or spoonsful of sugar and breast cancer risk did not differ within strata of BMI [4] and the other one found a nearly doubling risk with increased intake of desserts among more active (>7.54 MET-h/wk) compared with inactive women (0 MET-h/wk) and a stronger association among women with a lower BMI (<25 kg/m^2^) [20]. However, these analyses relied on different thresholds for stratification and did not assess associations by menopausal status, making comparison difficult.

Several biological mechanisms may explain the higher MD observed in this study with higher sweet items intake. Intake of sugar seems to have an effect on cellular proliferation in general [7] and also on breast tumor cells *in vitro*[8], and increases incidence of tumors in animal models [55,56]. Moreover, an *in vitro* study on breast tumor cells has shown that while fructose appears to increase cell invasion and migration, glucose seems to increase cell proliferation [8]. These observations suggest that intake of sweet items could enhance cellular proliferation in breast tissues and could increase MD. In humans, excessive sugar consumption is also associated with an increase in production of insulin [7,57] that, in turn, can increase insulin-like growth factor-I (IGF-I) [58,59], a growth factor that has mitogenic and anti-apoptotic effects on cells. These effects were seen in both normal and abnormal breast cells [60-62]. Wang *et al.* found that feeding mice with a high sucrose diet could promote the proliferation and tumorigenesis of intestinal epithelial cells by increasing insulin and IGF-I levels [63]. In fact, IGF-I levels have been positively associated with breast cancer risk [64-66] and MD [36,67,68], particularly among premenopausal women. Regarding insulin, a positive association with breast cancer risk has been shown for postmenopausal women [69]. However, the possible effect of insulin levels on MD has not yet been shown [70,71]. Another possible mechanism of action of sugar consumption is through its effect on circulating levels of 25-hydroxyvitamin D (25(OH)D), the principal circulating vitamin D metabolite. This mediated action is likely because sugar-sweetened beverages, particularly colas, have been shown to decrease levels of serum 25(OH)D in an animal model [72] and in a study involving children [73]. At a cellular level, vitamin D (revealed by the levels of 25(OH)D as its biomarker) has been found to have anti-proliferative and pro-apoptotic effects [74]. Furthermore, low levels of 25(OH)D were found to be associated with an increase in breast cancer risk and MD among premenopausal women [75-77]. All of these hypotheses need to be confirmed with additional studies.

### Strengths and limitations

This study has several strengths. Firstly, the size of the study sample is relatively large and the equal number of premenopausal and postmenopausal women allows stratified analyses with enough power. Secondly, the breast mammographic images are of great quality. Almost all mammograms were done with equipment (LORAD M4) accredited by the Canadian Association of Radiology in order to satisfy the high quality standards of the Quebec breast cancer screening program. Thirdly, all measures of MD were done using a computer-assisted method with a trained reader without any information on women in a short period of time. Furthermore, the reader’s reliability of readings was high. Thus, misclassification of MD is unlikely and, if present, it should not have biased our results. Fourthly, the FFQ used in this study was validated in several studies for its accuracy [40,41]. Finally, adjustment was made for factors known or suspected to be related to MD and for total caloric intake to control for most of the potential confounding biases. Since all of the women in this study were Caucasian, analyses were not adjusted for ethnicity.

This study has also some limitations. We aimed at clarifying the effect of consumption of sweet foods on MD because they contain a lot of sugar, but some of these foods also contain other nutrients, like fat, that may cause residual confounding. Thus, the association observed between sweet foods intake and MD among postmenopausal women could be due to nutrients other than sugar. However, the sugar-sweetened beverages group is mostly composed of sugar. Therefore, the association found with sugar-sweetened beverage intake and MD among premenopausal women supports our hypothesis that higher consumption of sugar can lead to higher MD. Misclassification in the frequency of consumption of food items is possible with the use of the FFQ. Participants have to give an estimation of the frequency of consumption of an item over the previous year. However, differential bias is unlikely because answers inaccuracies can hardly be related to MD and the misclassification can only lead to an underestimation of the effect. Furthermore, the cross-sectional design of this study does not allow causal interpretation and prospective studies are necessary to establish causality. We also cannot exclude that our findings may be due to chance because we evaluated several associations. Type I errors or false-positive results are therefore possible. Finally, it is p that power was insufficient for stratified analyses by BMI and physical activity in each menopausal group and therefore these results should be interpreted with caution.

## Conclusions

This study found that higher consumption of sweet foods among postmenopausal women and higher consumption of sugar-sweetened beverages among premenopausal women are associated with MD. Given the small number of studies that examined the relationship between intake of sweet items and MD, these associations need to be confirmed by other studies. The difference in MD found in our study might not be very high, but it may not be neglected in a global strategic plan to prevent breast cancer by reducing MD. Considering the worldwide increase in sugar consumption and all the health problems it is related to, it is important to continue research on this subject and begin to inform the public about the adverse effects of sugar consumption.

## Abbreviations

MD: Mammographic density; WHO: World health organization; FAO: Food and agriculture organization of the United Nations; FFQ: Food frequency questionnaire; BMI: Body mass index; MET-h/wk: Metabolic equivalent hours per week; OR: Odds ratio; IGF-I: Insulin-like growth factor; 25(OH)D: 25-hydroxyvitamin D.

## Competing interest

The authors declare that they have no competing interest.

## Authors’ contributions

CSD performed statistical analysis with input from CD, and wrote the first draft of the manuscript. ID assisted in the interpretation of findings and was involved in the critical revising of the manuscript. CD conceived the study, participated in statistical analysis, in the interpretation of findings, in the drafting and revision of the manuscript. All authors read and approved the final manuscript.

## Pre-publication history

The pre-publication history for this paper can be accessed here:

http://www.biomedcentral.com/1471-2458/14/554/prepub

## Supplementary Material

Additional file 1 Partial Spearman correlations of sugar-sweetened beverages and sweet foods intake with MD stratified by BMI and physical activity among all, premenopausal and postmenopausal women. Click here for file
