# Supplementary material for: Consumption of sweet foods and mammographic breast density: a cross-sectional study
Source: BMC Public Health. 2014 Jun 26;14:554. doi: 10.1186/1471-2458-14-554 (PMC4071328; doi:10.1186/1471-2458-14-554)
Supplement: Additional file 1 — Partial Spearman correlations of sugar-sweetened beverages and sweet foods intake with MD stratified by BMI and physical activity among all, premenopausal and postmenopausal women. [file 1471-2458-14-554-S1.docx]

| **Additional file 1.** Spearman correlations of sweet foods and sugar-sweetened beverages intakes with mammographic density stratified by BMI and physical activity among all, premenopausal and postmenopausal women | | | | | | | | | |
| --- | --- | --- | --- | --- | --- | --- | --- | --- | --- |
|  |  | **All women** | |  | **Premenopausal women** | |  | **Postmenopausal women** | |
| Groups of foods (servings per week) | n | Percent density (%) | Absolute density (cm^2^) | n | Percent density (%) | Absolute density (cm^2^) | n | Percent density (%) | Absolute density (cm^2^) |
|  |  | *r*(*P*) ^a^ | *r*(*P*) ^a^ |  | *r*(*P*) ^a^ | *r*(*P*) ^a^ |  | *r*(*P*) ^a^ | *r*(*P*) ^a^ |
| **Sweet foods^b^** | | | | | | | | | |
| BMI (kg/m^2^) |  |  |  |  |  |  |  |  |  |
| ≤25 | 711 | -0.009 (0.814) | -0.035 (0.358) | 438 | -0.018 (0.716) | -0.012 (0.801) | 273 | 0.006 (0.925) | -0.048 (0.447) |
| >25 | 803 | 0.008 (0.826) | 0.009 (0.810) | 325 | -0.010 (0.076) | -0.071 (0.219) | 478 | 0.085 (0.070) | 0.069 (0.139) |
| Physical activity (MET-h/wk) | | |  |  |  |  |  |  |  |
| ≤21 | 759 | 0.023 (0.544) | 0.007 (0.856) | 366 | -0.100 (0.064) | -0.070 (0.195) | 393 | 0.145 (0.005) | 0.089 (0.089) |
| >21 | 755 | -0.009 (0.812) | -0.020 (0.595) | 397 | -0.009 (0.857) | -0.013 (0.804) | 358 | -0.017 (0.756) | -0.015 (0.787) |
| **Sugar-sweetened beverages^c^** | | | | | | | | | |
| BMI (kg/m^2^) |  |  |  |  |  |  |  |  |  |
| ≤25 | 711 | 0.030 (0.434) | 0.030 (0.430) | 438 | 0.026 (0.601) | 0.071 (0.148) | 273 | -0.002 (0.972) | -0.023 (0.721) |
| >25 | 803 | 0.055 (0.128) | 0.050 (0.160) | 325 | 0.083 (0.149) | 0.101 (0.078) | 478 | 0.026 (0.577) | 0.016 (0.734) |
| Physical activity (MET-h/wk) | | |  |  |  |  |  |  |  |
| ≤21 | 759 | 0.080 (0.030) | 0.060 (0.103) | 366 | 0.079 (0.146) | 0.085 (0.117) | 393 | 0.042 (0.422) | 0.027 (0.604) |
| >21 | 755 | 0.038 (0.306) | 0.053 (0.150) | 397 | 0.025 (0.634) | 0.092 (0.074) | 358 | 0.027 (0.621) | 0.013 (0.816) |
| ^a^ Adjusted analyses for total caloric intake per day, age at mammography, body mass index, waist-to-hip ratio, menopausal status (for total women only), age at menarche, number of full-tem pregnancies, age at first full-term pregnancy, duration of use of hormonal contraceptive, duration of use of hormonal therapy, alcohol intake, physical activity, family history of breast cancer, personal history of biopsy, smoking status, education and breastfeeding.  ^b^ Including ice cream, chocolate, candy with chocolate, candy, homemade cookie, commercial cookie, brownie, donut, homemade cake, commercial cake, homemade pie, commercial pie, other homemade pastries and other commercial pastries  ^c^ Including cola with sugar, cola with sugar but caffeine free, other carbonated beverage with sugar and sweet fruits juice  **Abbreviations:** r = correlation, BMI = body mass index, MET-h/wk = Metabolic equivalent hours per week | | | | | | | | | |
